# Cimetidine increases survival of colorectal cancer patients with high levels of sialyl Lewis-X and sialyl Lewis-A epitope expression on tumour cells

**DOI:** 10.1038/sj.bjc.6600048

**Published:** 2002-01-21

**Authors:** S Matsumoto, Y Imaeda, S Umemoto, K Kobayashi, H Suzuki, T Okamoto

**Affiliations:** Department of Surgery, Second Teaching Hospital, School of Medicine, Fujita Health University, 3-6-10 Otohbashi, Nakagawa-ku, Nagoya 454-8509, Japan; Department of Molecular Genetics, Nagoya City University Medical School, 1 Kawasumi, Mizuho-cho, Mizuho-ku, Nagoya 467-8601, Japan

**Keywords:** colorectal cancer, cancer metastasis, sialyl Lewis antigens, cell adhesion, cimetidine

## Abstract

Cimetidine has been shown to have beneficial effects in colorectal cancer patients. In this study, a total of 64 colorectal cancer patients who received curative operation were examined for the effects of cimetidine treatment on survival and recurrence. The cimetidine group was given 800 mg day^−1^ of cimetidine orally together with 200 mg day^−1^ of 5-fluorouracil, while the control group received 5-fluorouracil alone. The treatment was initiated 2 weeks after the operation and terminated after 1 year. Robust beneficial effects of cimetidine were noted: the 10-year survival rate of the cimetidine group was 84.6% whereas that of control group was 49.8% (*P*<0.0001). According to our previous observations that cimetidine blocked the expression of E-selectin on vascular endothelium and inhibited the adhesion of cancer cells to the endothelium, we have further stratified the patients according to the expression levels of sialyl Lewis antigens X (sL^x^) and A (sL^a^). We found that cimetidine treatment was particularly effective in patients whose tumour had higher sL^x^ and sL^a^ antigen levels. For example, the 10-year cumulative survival rate of the cimetidine group with higher CSLEX staining, recognizing sL^x^, of tumours was 95.5%, whereas that of control group was 35.1% (*P*=0.0001). In contrast, in the group of patients with no or low levels CSLEX staining, cimetidine did not show significant beneficial effect (the 10-year survival rate of the cimetidine group was 70.0% and that of control group was 85.7% (*P*=n.s.)). These results clearly indicate that cimetidine treatment dramatically improved survival in colorectal cancer patients with tumour cells expressing high levels of sL^x^ and sL^a^.

*British Journal of Cancer* (2002) **86**, 161–167. DOI: 10.1038/sj/bjc/6600048 www.bjcancer.com

© 2002 The Cancer Research Campaign

## 

We demonstrated in a previous randomized control study that cimetidine, a histamine type 2 receptor antagonist, was significantly advantageous in increasing the disease-free period and survival of these patients (Matsumoto, 1995). Cimetidine was given to colorectal cancer patients receiving 5-fluorouracil (5-FU) after operation with the aim of reducing appetite loss and reflux esophagitis. Two other study groups reported similar advantageous effect of cimetidine on colorectal cancer patients (Adams and Morris, 1994; Svendsen *et al*, 1995). Furthermore, treatment with cimetidine was reported to be beneficial for patients with gastric cancer (Tonnesen *et al*, 1988), melanoma (Creagan *et al*, 1985; Hellstrand *et al*, 1994) or renal cell cancer (Sagaster *et al*, 1995).

Several studies have suggested various mechanisms underlying the beneficial effect of cimetidine on cancer patients, such as the following: (i) reversal of the pharmacological activity of histamine, tumour growth promoter by blocking histamine receptors on cancer cells (Adams *et al*, 1994; Reynolds *et al*, 1996) or affecting histamine metabolism (Garcia-Caballero *et al*, 1994); (ii) acting as an antioxidant, thus inhibiting tumour growth (Kimura *et al*, 1986) and (iii) augmentation of anticancer immune reactivity through receptor antagonism of circulatory suppressor T cells (Kumar, 1990), prevention of postoperative alterations of lymphocyte subpopulations (Hansbrough *et al*, 1986), or by maintenance of natural killer cell activity (Katoh *et al*, 1996). In our study, we found that cimetidine could block the expression of E-selectin on the surface of human umbilical vein endothelial cells, thus blocking the tumour cell adhesion to endothelium and preventing the liver metastasis in nude mice model (Kobayashi *et al*, 2000). Such findings were not known when we had planned this prospective randomized control study. But now, it is known that sialyl Lewis-X (sL^x^) and sialyl Lewis-A (sL^a^) antigens are ligands to E-selectin, and sL^x^ and sL^a^ expressed on cancer cells mediate adhesion of the cancer cells to vascular endothelial cells expressing E-selectin (Phillips *et al*, 1990; Takada *et al*, 1991, 1993). The adhesion of cancer cells to vascular endothelial cells is a key step in invasion and metastasis of cancer cells (Hoff *et al*, 1989, 1990; Nakamori *et al*, 1993). Therefore, we decided to classify the subjects according to the level of expression of sL^x^ and sL^a^ on cancer cells and we investigated 10-year survival that was the major objects of this clinical study. We examined whether the effect of cimetidine on cancer patients was correlated with the degree of expression of sL^x^ and sL^a^ on tumour cells in 61 colorectal cancer patients in our randomized control study (Matsumoto, 1995). Treatment with cimetidine markedly reduced the incidence of metastasis and significantly increased survival during a follow-up period of more than 10 years in patients whose tumour cells expressed sL^x^ and sL^a^ epitopes at increased levels.

## MATERIALS AND METHODS

### Patients

This randomized control study was performed on colorectal cancer patients in a multicentre clinical trial of cimetidine. The clinical trial was conducted through the collaboration of 15 institutions in Japan listed at the end of the text. The coordination centre for the trial was the Department of Surgery, Second Teaching Hospital, School of Medicine, Fujita Health University. It was carried out with the approval of Fujita Health University Ethical Committee. A total of 72 patients who were diagnosed colorectal cancer by histological examination and had a primary tumour of T_2_ or T_3_ were enrolled after excluding patients who previously received chemotherapy, radiotherapy or immunotherapy and those who had multiple cancers or severe complications. Out of the 72 selected, patients who did not undergo curative resection (two patients), those who did not receive adequate drug administration (three patients), and whose disease stage was considered inappropriate for the trial (three patients) were further considered ineligible. These ineligible patients were equally distributed between the treatment groups and were excluded from the analysis. All patients were randomly allocated and there were none lost to follow-up. All 64 eligible patients gave informed consent to take part in the clinical trial and were enrolled for the trial from March 1990 to April 1992 (Table 1

**Table 1:**
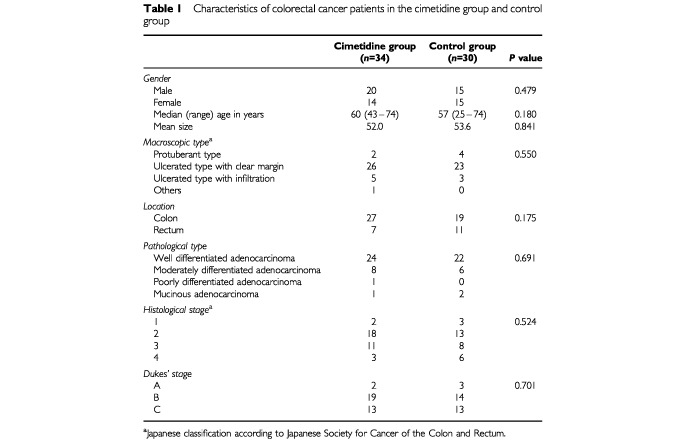
Characteristics of colorectal cancer patients in the cimetidine group and control group

). The patients were followed up until the end of May 2000, with a mean follow-up term of 10.7 years. During the follow-up period, the patients were checked at least twice a year for occurrence of metastasis as well as for blood chemistry, X-ray, ultrasonography and computed tomography. Survival was the primary endpoint. Time to recurrence (disease-free period) was also assessed.

### Treatment of the patients

The 64 patients were classified into two groups consisting of 34 and 30 for the treatment and control group, respectively. There were no differences in age, sex, clinical characteristics and macroscopic shape, size, location, stage and pathological type of the cancer between the two groups (Table 1). All patients received curative resection of the cancer and within 24 h of the resection, were intravenously injected with 8 mg m^−2^ of mitomycin C (Kyowa Hakko, Inc, Tokyo, Japan). The patients in the cimetidine group (*n*=34) were given 800 mg day^−1^ of cimetidine orally (SmithKline Beecham, Co., Tokyo, Japan) together with 200 mg day^−1^ of 5-FU orally (Kyowa Hakko, Inc.), while patients in the control group (*n*=30) received 5-FU alone. Treatment for both groups started 2 weeks after the operation and was given for 1 year.

### Immunostaining of sL^x^ and sL^a^ on cancer cells

Cancer tissues, which had been resected by the curative operation and embedded in paraffin, were used for immunostaining of sL^x^ and sL^a^. A total of 61 specimens were processed since we lost the specimens of two patients in the treatment group and of one patient in the control group. Immunostaining was performed by the avidin biotin complex method. Three different anti-sL^x^ monoclonal antibodies (mAbs), CSLEX (Signet Lab., Dedham, MA, USA) (Fukushima *et al*, 1984), KM93 (Kyowa Hakko, Inc) (Shirata *et al*, 1987) and FH6 (Otsuka Pharmaceutical Co., Osaka, Japan) (Fukushi *et al*, 1984) were used. The CA19-9 mAb (CIS Bio International, Cedex) (Charpin *et al*, 1982) was used as the anti-sL^a^ mAb. Cancer tissues in paraffin blocks were sectioned. The sections were deparaffinized in xylene, dehydrated through graded concentrations of ethanol and washed with distilled water. After treatment of the sections with bovine serum albumin to block nonspecific binding of the primary mAb, the sections were incubated in either one of the anti-sL^x^ mAbs or the anti-sL^a^ mAb for 2 h. The sections were rinsed with phosphate buffered saline (PBS), and then incubated in biotinylated anti-mouse immunoglobulin serum (Vector Lab, Burlingame, CA, USA) for 30 min. After washing with PBS, the sections were immersed in 0.3% (wt/vol) hydrogen peroxide in absolute methanol for 20 min to block endogenous peroxidase. The sections were again washed with PBS and incubated in avidin-conjugated horseradish peroxidase (Vector Lab) for 30 min, and then washed with PBS. Finally, the sections were incubated in peroxidase substrate solution until the desired stain intensity had developed (1 to 5 min). After washing with distilled water, the sections were counterstained with hematoxylin, dehydrated in ethanol, washed in xylene and mounted.

### Determination of degree of sL^x^ and sL^a^ expression on cancer cells

By microscopic observation of the predominant area of the cancer tissue in the immunostained section, the percentage of positively stained cancer cells was calculated. Two pathologists observed the specimens and determined degrees of sL^x^ and sL^a^ expression. The degrees of expression of sL^x^ and sL^a^ based on the percentage of positively stained cancer cells were presented as follows: level 0, no stained cancer cells; level 1, less than 5% cancer cells stained; level 2, 5–70% cancer cells stained; level 3, 71% or more cancer cells stained. We defined the classification of patients according to the degree of sLe^x^ and sLe^a^ expression as such based on our observations that the number of cases with tumours exhibiting low-positively staining cancer cells in each category was very few. For example, among the cases with level 2 for CSLEX, 19 out of 21 cases (90%) exhibited the tumour more than 30% positively stained cancer cells and similarly, among the cases with level 2 expression of KM93, all 24 cases (100%) exhibited the tumour with over 60% positively stained cancer cells. In addition, 95% of the cases defined as level 2 for CA19-9 exhibited over 30% of positively stained cancer cells. There was no case exhibited where the tumour tissue with less than 10% stained cancer cells with any of the sialyl Lewis antigens.

### Statistical analyses

Cumulative survival rate was calculated by the Kaplan-Meier method. Statistical significance of the difference in the survival rate of patients between two categories was evaluated by the log rank test or the generalized Wilcoxon test. Differences in metastasis frequency were calculated by Fisher's exact *t*-test. A *P* value of <0.05 was considered significant.

## RESULTS

### Cimetidine treatment increases survival of colorectal cancer patients

**Figure 1:**
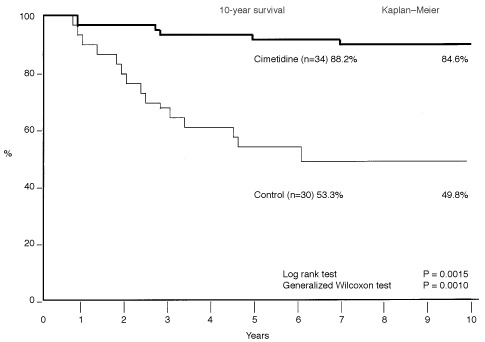
Effect of cimetidine on the survival of patients with colorectal cancers. Patients who were treated with cimetidine and 5-FU (‘cimetidine’ group) and 5-FU alone (‘control’ group) were compared by Kaplan-Meier method. The cumulative 10-year survival rate of the cimetidine group (*n*=34) was 84.6%, whereas that of control group (*n*=30) was 49.8% (*P*=0.0015 by log rank test and *P*=0.0010 by generalized Wilcoxon test).

Among the 64 patients with curative operation, 34 cases were classified into the cimetidine group (800 mg day^−1^ of cimetidine orally for 1 year) and 30 cases served as the control group (without cimetidine treatment). All the patients were treated with 5-FU (200 mg day^−1^) for 1 year. In order to evaluate the effect of cimetidine, the survival rates were compared between these two groups (Figure 1

). The cumulative 10-year survival rate of the cimetidine group (*n*=34) was 84.6%, whereas that of control group (*n*=30) was 49.8% (*P*=0.0015 by log rank test and *P*=0.0010 by generalized Wilcoxon test).

**Figure 2:**
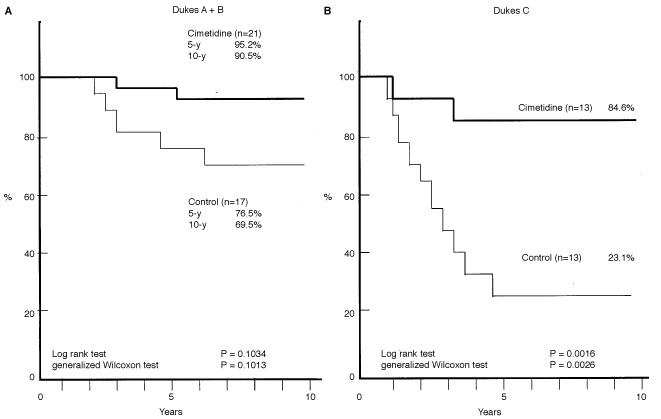
Effect of cimetidine on the survival of patients with colorectal cancer according to the Dukes classification. Dukes A and B, localized cancer limited to mucosa and submucosa (**A**) and extending through serosa without lymph node metastasis (**B**). Dukes C, cancers involving regional lymph nodes. Note that the beneficial effects of cimetidine were greater in patients with Dukes C: the cumulative 10-year survival rate of the cimetidine group (*n*=13) was 84.6%, whereas that of control group (*n*=13) was 23.1% (*P*=0.0016 by log rank test and *P*=0.0026 by generalized Wilcoxon test).

We then evaluated the effects of cimetidine according to the clinical stage of colorectal cancer. As shown in Figure 2

, the effect of cimetidine on the survival of patients with involvement of regional lymph nodes, thus classified as Dukes C, was remarkably significant. The cumulative 10-year survival rate of the cimetidine group of Dukes C patients (*n*=13) was 84.6%, whereas that of the control group (*n*=13) was 23.1% (*P*=0.0016 by log rank test; *P*=0.0026 by generalized Wilcoxon test). In contrast, the effect of cimetidine on the survival of patients at Dukes A or B classification were not statistically significant, although there was some beneficial tendency for the cimetidine group (the cumulative 10-year survival rate of the cimetidine group (*n*=21) was 90.5%, whereas that of the control group (*n*=17) was 69.5% (not significant both by log rank and the generalized Wilcoxon tests).

### Cimetidine reduces frequency of metastasis in colorectal cancer patients

The incidence of new metastasis in colorectal cancer patients over a period of 10 years after curative resection of the tumour was compared between the two study groups; the cimetidine group (treated with cimetidine and 5-FU) and a control group (treated with 5-FU alone). In the cimetidine group (*n*=34), eight metastases occurred in seven patients, whereas in the control group (*n*=30), 23 metastases occurred in 16 patients. The frequency and location of metastases in these colorectal cancer patients are shown in Table 2

**Table 2:**
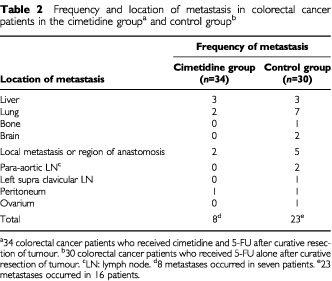
Frequency and location of metastasis in colorectal cancer patients in the cimetidine group^a^ and control group^b^

. Overall incidence of metastasis was significantly reduced in the cimetidine (*P*=0.0060 by Fisher's *t*-test).

### Cimetidine treatment increases survival of colorectal cancer patients with high-level sL^x^ or sL^a^ epitope expression on tumour cells

Expression of sL^x^ and sL^a^ on cancer cells was determined by immunostaining of tumour tissues with anti-sL^x^ and anti-sL^a^ monoclonal antibodies (mAbs). The patients were grouped according to the level of expression of sL^x^ or sL^a^ on their tumour cells as described in Materials and methods.

**Figure 3:**
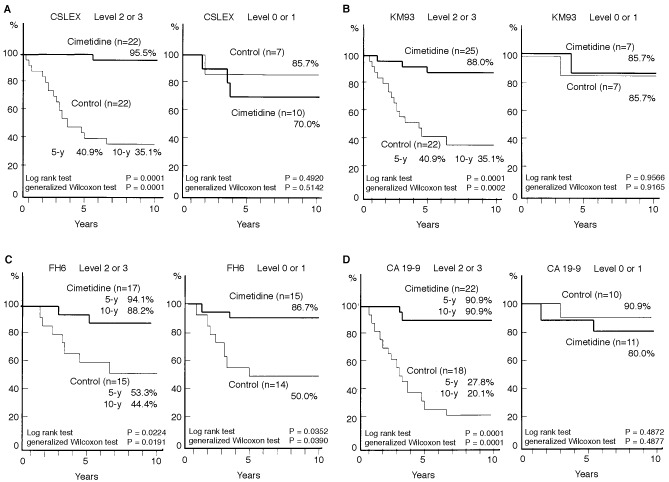
Effect of cimetidine on cumulative survival rates in the colorectal cancer patients. The tumour tissues from individual patients obtained during curative operations were stained by mAbs to sL^x^ antigen, including CSLEX (**A**), KM93 (**B**), FH6 (**C**) and sL^a^ antigen, CA19-9 (**D**). The level of these epitope expression were semi-quantitated upon microscopic examination by two experienced pathologists: level 1, less than 5% cancer cells stained; level 2, 5–70% cancer cells stained; level 3, 71% or more cancer cells stained. The survival rates of patients with cimetidine treatment (‘Cimetidine’) or without cimetidine treatment (‘Control’) were compared using Kaplan-Meier method. The number of patients (‘*n*’) in each category was indicated in the figure. The statistical significance was evaluated by log rank test and generalized Wilcoxon test.

The effect of cimetidine on the survival of patients was evaluated with regard to the level of sL^x^ and sL^a^ antigens. The cumulative survival rates of patients, with or without cimetidine, based on the CSLEX epitope expression were demonstrated in Figure 3A

. In patients with high CSLEX expression (levels 2 or 3), the cumulative 10-year survival rate of the cimetidine group (*n*=22) was 95.5% whereas that of control group (*n*=22) was 35.1% (*P*=0.0001 by log rank test and generalized Wilcoxon test). In contrast, patients with no (level 0) or low (level 1) CSLEX expression, the cumulative 10-year survival rate of the cimetidine group (*n*=10) was 70.0 and 85.7% for the control group (*n*=7), (not significant by log rank and generalized Wilcoxon tests).

Cimetidine had a similar positive effect on the survival rate of patients with high-level KM93 sL^x^ or CA19-9 sL^a^ epitopes expression on their tumours. The results of survival based on KM93 mAb expression were shown in Figure 3B. In patients with high KM93 expression, the cumulative 10-year survival rate of the cimetidine group (*n*=25) was 88.0%, whereas that of the controls (*n*=22) was 35.1% (*P*=0.0001, log rank test; *P*=0.0002, generalized Wilcoxon test). In contrast, in patients with low level or no KM93 expression, the cumulative survival rates were equal for the cimetidine group (*n*=7) and the control group (*n*=7) (85.7% for both).

Similarly, the results of survival according to CA19-9 mAb expression were shown in Figure 3D. In patients with high level of sL^a^ expression, the cimetidine treatment was effective: the cumulative 10-year survival rate for the cimetidine (*n*=22) and the control (*n*=18) groups were 90.9 and 20.1%, respectively (*P*=0.0001). However, for patients with no or low level sL^a^ expression, there was no beneficial effect of the cimetidine treatment: cumulative 10-year survival rate for the cimetidine group (*n*=11) was 80.0 and 90.9% for the control group (statistically not significant).

The effect of cimetidine on patients' survival was not always correlated with the level of sL^x^ and sL^a^ epitopes. As shown in Figure 3C, using FH6 mAb recognizing one of the sL^x^ epitopes, the beneficial effect of cimetidine treatment was evident irrespective of the level of FH6. In patients with high-level FH6 expression, the cumulative 10-year survival rate of the cimetidine (*n*=17) and the controls (*n*=15) groups were 88.2 and 44.4%, respectively (*P*=0.0224, log rank test; *P*=0.0191, generalized Wilcoxon test). In addition, even in patients with low level or no FH6 expression, the beneficial effects of cimetidine on the patients survival were significant: the cumulative 10-year survival rate for the cimetidine (*n*=15) and the control (*n*=7) group were 86.7 and 50.0% (*P*=0.03520, log rank test; *P*=0.0390, generalized Wilcoxon).

## DISCUSSION

Adhesion of cancer cells via their sL^x^ or sL^a^ antigen to E-selectin on vascular endothelium is considered to lead to metastasis. Based on our recent observations that cimetidine blocked *in vitro* expression of E-selectin on the surface of vascular endothelial cells as reported by Kobayashi *et al*. (2000), we examined in this study whether the beneficial effect of cimetidine on colorectal cancer patients was dependent on the degree of expression of sL^x^ and sL^a^ on the tumour cells.

In the control group patients (treated with 5-FU alone after curative operation) of these studies, we noticed that the patients with tumours expressing sL^x^ and sL^a^ antigens at higher levels showed a markedly higher frequency of metastasis and a significantly lower survival rate, indicating its aggressive characteristics. These results largely agree with Nakamori *et al* (1993) who reported that colonic cancers expressing the sL^x^ epitopes recognized by FH6 mAb at higher levels were more malignant. In our study, however, malignancy of the cancer was related to the expression of sL^x^ epitopes recognized by CSLEX and KM93 mAbs, but not to the expression of sL^x^ epitope recognized by FH6 mAb. The reason for this discrepancy might depend on different binding property of these three monoclonal antibodies to the epitopes of tumour cells (Charpin *et al*, 1982; Dohi *et al*, 1993). If the epitope, which was recognized with FH6 mAb, reacted with E-selectin with high intensity, cimetidine could inhibit sticking of the tumour cells to endothelial cells. Our results also indicated that colorectal cancer expressing the sL^a^ epitope recognized by the CA19-9 mAb at higher levels appeared to be as aggressive as those expressing the sL^x^ epitope.

The fact that elevated expression of the sL^x^ epitopes recognized by the CSLEX or KM93 mAbs and the sL^a^ epitope recognized by the CA19-9 mAb were associated with more aggressive nature of colorectal cancer malignancy suggested the importance of these epitopes as ligands for E-selectin. To define the sL^x^ and sL^a^ epitopes that were important E-selectin ligands, Srinivas *et al* (1996) determined the blocking activity of several anti-sL^x^ and anti-sL^a^ mAbs on the adhesion of human colonic cancer cell lines to human vascular endothelial cells *in vitro*.

They reported that anti-sL^x^ CSLEX and KM93 mAbs did not block adhesion, probably because of the very low levels of sL^x^ expression in the cancer cell lines detected by Western blotting, and that the anti-sL^a^ CA19-9 mAb blocked adhesion of one cancer cell line but not that of another cancer cell line. Phillips *et al* (1990) demonstrated that anti-sL^x^ CSLEX mAb blocked adhesion of a human promyelocytic cell line to human endothelial cells. These results indicated the complexity of expression and function of sL^x^ and sL^a^ epitopes on the established cell lines. However, it is conceivable that down regulation of E-selectin, a ligand for sL^x^ and sL^a^ antigens, by cimetidine can block some of the malignant phenotype of cancer as predicted by our recent study (Kobayashi *et al*, 2000).

The present study clearly demonstrated that the beneficial effect of cimetidine given together with 5-FU after a curative operation on colorectal cancer patients depended on the degree of expression of the sL^x^ and sL^a^ epitopes on tumour cells. Treatment with cimetidine markedly reduced the frequency of metastasis and significantly increased survival rate in the patients whose cancer cells expressed higher levels of the sL^x^ and the sL^a^ epitopes, but not in the patients whose cancer cells expressed none or lower levels of these epitopes, although such a cancer was considered to be less aggressive. Colorectal cancer cells expressing higher levels of sL^x^ and sL^a^ should adhere easily to vascular endothelium expressing E-selectin, and this would then result in metastasis, a marker of malignancy. If cimetidine given to the patients blocked the expression of E-selectin on vascular endothelial cells, even malignant colorectal cancer cells expressing higher levels of sL^x^ and sL^a^ would not be able to adhere to such vascular endothelial cells. In this situation, the frequency of metastasis in the patients would be reduced and the survival rate of the patients increased. Taken together, these results suggested a mechanism underlying the beneficial effect of cimetidine on colorectal cancer patients, presumably by blocking the expression of E-selectin on vascular endothelial cells and inhibiting the adhesion of cancer cells.

These results with cimetidine suggest that the cognate interaction of sL^x^ and sL^a^ antigens with E-selectin provides a novel target for prevention of cancer progression. It is likely that cimetidine treatment may also be effective for a range of other sL^x^- and sL^a^-expressing tumours, such as oesophageal, gastric, pulmonary, pancreatic, biliary tract, uterine, ovarian and breast cancer. Our study was so small a number scale that further large-scale study should be investigated to assess the effect of cimetidine to colon cancer with high expression of sialyl Lewis antigens.
